# P300 Auditory Evoked Potential Latency In Elderly

**DOI:** 10.1590/S1808-86942010000300003

**Published:** 2015-10-20

**Authors:** Maria José Santos Cóser, Pedro Luis Cóser, Fleming Salvador Pedroso, Rafaele Rigon, Elenara Cioqueta

**Affiliations:** 1 MSc in Human Communications Disorders – Federal University of Santa Maria -UFSM, ENT at the UFSM; 2 PhD in Human Communications Disorders – UNIFESP, Associate Professor of Otolaryngology – UFSM; 3 PhD in Medicine – UFRGS, Professor of Pediatrics and Neurology – UFSM; 4 MSc in Human Communications Disorders – UFSM, Speech and Hearing Therapist of the ENT Cóser Clinic; 5 MSC in Human Communications Disorders – UFSM, Speech and Hearing Therapist of the ENT Cóser Clinic. Federal University of Santa Maria

**Keywords:** elderly, hearing loss, auditory evoked, potentials, p300 event related potentials

## Abstract

Auditory cortical disorders in the elderly can be assessed by the P300. The lack of contemporary reference values of P300 latency in healthy elderly motivated this study.

**Aim:** To estimate the effect of age on P300 latency in a group of elderly.

**Methods:** We studied 62 elderly patients with pure tone thresholds up to 40 dB HL at the frequencies of 1000 and 2000 Hz, divided into groups according to age (60-64, 65-69 and 70-74 years). Were assessed by the P300 latency in response to the rare stimulus of 2000 Hz and 1000 Hz frequencies, both in the intensity of 80 dB HL.

**Study design:** clinical, cross-sectional observational individual compared prospective.

**Results:** The latency in Group 60 was 337.26 ms (SD 11.31) in Group 65 was 351.86 ms (SD 29.05) and in Group 70 it was 370.19 ms (SD 23.40). The linear regression of the values of P300 latency showed an increase of 2.85 ms per year of age. The statistical analysis showed that the results were significant.

**Conclusion:** The P300 latency increases with age at a rate of 2.85 ms per year between the ages of 60 and 74 years.

Send correspondence to: Maria José Santos Cóser – Rua Duque de Caxias 1668/304 Centro 97015-190 Santa Maria RS Brasil.

Paper submitted to the BJORL-SGP (Publishing Management System – Brazilian Journal of Otorhinolaryngology) on January 20, 2008; and accepted on February 25, 2010. cod. 5686

## INTRODUCTION

A growing number of elderly people who listen well and who complain they do not understand speech in environments where other people are speaking, or there is music or noise present, have sought otorhinolaringologists^1^. Hearing loss in high frequencies, when present, explains some of these complaints, but in recent years an additional factor attributed to functional changes in the cerebral cortex and associated with aging has been recognized^1^,^2^. Neurochemical and structural changes are already demonstrably involved in this process^3^, ^4^, ^5^. Some of these people have a perfectly normal tone audiometry or with mild losses that would not justify the difficulty in understanding speech. To make a proper diagnosis of cerebral dysfunction in hearing is a challenge to modern audiology. Studies on auditory behavioral tests that assess the Auditory Central Nervous System (ACNS) have been used for this purpose, but still in a much smaller scale than those which evaluate dysfunctions in peripheral hearing^6^.

Sutton et al.^7^ in 1965 published a study of positive potentials with latency around 300 milliseconds (ms) evoked by the presentation of a “rare” tonal stimulus; unexpectedly presented in a sequence of “frequent” tonal stimuli. The individual being examined should pay attention to rare stimuli and perform a task every time it was perceived. The task could be to count the number of rare stimuli perceived. This potential lies beyond the compulsory stages of sensory processing and has been called endogenous, since its generation is dependent on voluntary action of the individual and not just passive perception of sound stimuli that are considered “exogenous”. These potentials are associated with active processes such as attention, perception, memory and cognition. Since the latency in normal young adults is close to 300 ms, the name P300 was adopted.

Currently it is considered that the P300, among other long latency auditory evoked potentials (LLAEP), plays a key role in audiology, for its ability to pick up electrical potentials generated in the ACNS-related cognition, without using invasive techniques, represents a unique diagnosis method which provides a spatial-temporal window through which one can understand the brain processes underlying auditory perception and processing^8^. The ability to assess central auditory processing disorders with P300 is recent and promising as demonstrated by the work of Jerger and Lew1 in 2004. They observed that P300 latency was significantly increased in elderly subjects with central auditory processing disorders detected by behavioral dichotic tests when compared with latency observed in elderly patients with presbycusis and normal behavioral dichotic tests. This assessment becomes even more important in the emergence of effective techniques for treating these disorders, which require a detailed diagnosis of the cortical dysfunction^9^.

Since the P300 latency increase occurs physiologically after the age of 15^10^, ^11^, ^12^, ^13^, age should be taken into account when interpreting the values obtained from different age groups. The international literature on this subject is concentrated in the 80s. At that time, individuals over 45 years were considered elderly and the studies involved a small number of individuals over 70. Controversial results in the values of P300 latency from these studies in “elderly” were obtained by a lack of standardization of auditory stimuli and tasks involved in responding to the test^14^, as well as by factors inherent to this age range^15^. Considering the above, it seems appropriate to establish P300 latency updated benchmarks in order to compare with values obtained from groups of individuals suspected of presenting auditory processing disorders (APD).

This study aims at establishing the effects of age on P300 latency in a population of healthy elderly people aiming at helping to establish these reference values.

## MATERIALS AND METHODS

This study is set within a paradigm of quantitative research in the field with data collection and descriptive-exploratory analysis characterized as a compared individual cross-sectional observational contemporary study; advanced age was the independent outcome variable and the main outcome variable was the P300 latency^16^. It was been carried out after obtaining the values of the 300ms Long Latency Auditory Evoked Potential (P300) in a group of elderly participants in the project “Elderly Swimming and Health” of the Integrated Center for Study and Support for Third Age (NIEATI) of the institution where the study was conducted. The main activity of this project is the practice of physical exercises once a week.

The study group consisted of individuals aged between 60 and 74 years (“young elderly” according to the WHO) participants in the NIEATI project. 253 seniors participated in the project (during the 2007 school year).

For inclusion in the study subjects should sign a term of informed consent agreeing to participate in it, present normal otoscopy, self-assess his/her hearing as very good or good enough, have enough education in order to understand and go through the test, make tonal auditory threshold in the frequencies 1000 and 2000 Hz equal to or below 40 dB HL and Percentage Index of Speech Recognition (SDT) higher than 80% for both sides and P300 latency between 250 and 500 ms; for positive peaks with values outside this range of latency can not be called P300 according to Pfefferbaum et al.^17^ (1984).

We took off from the study those subjects who had tone threshold above 40 dB HL in the frequencies of 1000 and 2000 Hz in either ear, SDT below 80% in either ear, history of stroke, head trauma, brain tumors, senile dementia, schizophrenia, aphasia, chronic renal failure, alcoholism, multiple sclerosis, Parkinson's disease, Huntington's disease, HIV positive, Alzheimer's disease, those who refused to participate in the project, defaulted on evaluations, used psychoactive drugs, otoscopy showed signs of impairment of anatomical integrity of the external ear canal and/or of the tympanic membrane.

The 62 seniors who formed the study group matched the inclusion and exclusion criteria for the study. The Commission on Teaching and Research and Ethics Committee of the Center for Health Sciences from the institution where the study was held approved it under protocol number 095/04 Resolution 196/96 of the National Research Ethics Committee. The director of NIEATI signed the Authorization for this institutional research, having been informed of the goals by the same researcher. After signing the Authorization Term, we established the days and times for collection of data which was first done at the medical clinic of the Center for Physical Education by the researcher or a student from the Medical School of the institution where the research was performed. The goal of the study was reported and the elderly were invited to join it on the day they came to the medical examination required to practice exercises. Those who accepted the invitation signed a warrant for participation in it through an informed consent form.

The study had three distinct stages:

– to start with, all seniors who had done their medical examination on Tuesdays and Wednesdays by medical students were individually submitted to the self-assessment of hearing questionnaire (Appendix A).
– as a second step, those seniors who self-assessed their hearing as very good or good, went to the University Hospital of the institution where otoscopy, pure tone audiometry and speech audiometry were done. These assessments were made by the researcher and two speech therapists.
– In a third step, those elderly people whose hearing tests met the inclusion and exclusion criteria underwent the P300 test. Anamnesis was geared towards identifying factors for sample exclusion, such as inadequate schooling for testing, the presence of neuropsychological disorders and use of psychoactive drugs, held simultaneously with the hearing self-assessment questionnaire (Appendix A). We performed ENT evaluation before each audiometry. Cerumen was removed from the individuals carrying it and those with changes seen during otoscopy were taken off the study.

The audiometries took place in a soundproof booth. We used the digital AMPLAID 315 audiometer to obtain the pure tone thresholds by air conduction, in the frequency range of 250-8000 Hz for bone conduction thresholds in the frequencies of 500-4000 Hz, the SDT for monosyllabic lists and the recognition threshold speech (LRF) with disyllabic lists.

The purpose of this evaluation was to determine which subjects had pure tone thresholds in 1000 and 2000 Hz equal to or better than 40 dB HL and SDT equal to or greater than 80%.

**APENDIX A- HEARING SELF-ASSESSMENT QUESTIONNAIRE**

Name:...............................

Date of Birth:......................

Male ( ) Female ( )

Telephone #:

My hearing is:

() very good

() good

() bad

() very bad

In a quiet environment, I:

() Understand it very well what people tell me

() Understand it well what people tell me

() Misunderstand what people tell me

() Understand it very poorly what people tell me

In a noisy environment, I:

() Understand it very well what people tell me

() Understand it well what people tell me

() Misunderstand what people tell me

() Understand it very poorly what people tell me

My hearing:

() does not impair my quality of life

() impairs a little my quality of life

() impairs a lot my quality of life

These frequencies were chosen for their great importance in speech perception and for the same reason, we used the same stimuli for the P300 test. Those subjects with thresholds up to 40 dB HL were included in the study, because in this age group we rarely find normal thresholds.

We carried out the examination of auditory P300 with a CONTRONIC device used for evaluating electrophysiological hearing. The individual was put on a reclining chair to feel as comfortable as possible. We used abrasive paste to clean the vertex skin, high frontal midline and right and left mastoids. After deploying the conductive electrolyte paste, we fixed the electrodes to the place mentioned with micropore tape. The electrode impedance was maintained 05 K ohm (most often below 03 K ohm) with a maximum difference of 02 K ohm between them. The patients were told that the test was estimated to take 30 minutes and the individual was asked to keep his gaze fixed to a certain point in front of them and to remain as still as possible during the test. The individuals were told on what to do – a mental count of the rare stimuli – and an initial training was done with the presentation of some stimuli for the individual to understand well the test dynamics. At the end of a series of 300 stimuli the individual should properly inform the number of rare stimuli counted, and up to 10% of errors was accepted for the test to be considered valid.

A sequence of acoustic stimuli was binaurally presented through headphones, with two signals of the same intensity (80 dB HL) and different frequencies (1000 and 2000 Hz) in the form of tone bursts with 50 ms duration, with a 30 ms plateau of 30 ms and 10 ms of rise and fall times. Within the sequence, the frequent stimulus was triggered (1000 Hz) in 80% of the time, and the rare stimulus (2000 Hz) was randomly triggered 20% of the time between frequent stimuli.

**Figure 1:**
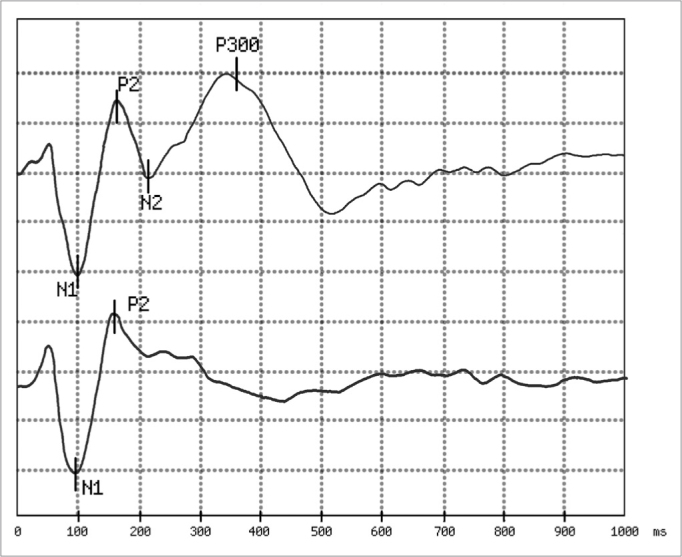
Example of answers obtained: In the first trace, obtained in response to a rare stimulus (a 2,000 Hz pure tone with an 80 dB HL intensity), we observe the presence of peaks N1 and P2 passively generated and peaks N2 and P300 generated by the mental count of the stimuli. In the second trace, obtained in response to a frequent stimulus (a 1,000 Hz pure tone with 80 dB HL intensity), we see only peaks N1 and P2 which are not associated with the task of counting sound stimuli. The analysis time was 1,000 ms and the sensitiveness of 5 microvolt per division. In this example, the P300 latency was 351 ms.

We recorded the electrophysiological responses in two simultaneous recording channels. One channel recorded the responses between the vertex electrode and that of the right mastoid and the other between the vertex and left mastoid. The signal mediator computer stored separately the appearance of responses for rare and frequent stimuli, using 1000ms window, 01-20 Hz filtered amplification, and a 0.8 pps repetition rate as depicted on Fig. 1. P300 was determined as the positive peak between 250 and 500 ms latency, according to recommendations of Pfefferbaum et al.^17^ (1984), observed in the curve where the answers were added to the rare stimuli.

Two sets of 300 stimuli (240 frequent and 60 rare) were presented in order to check for response reproducibility. In some cases, in which the P300 was not sufficiently defined, a third series was presented.

**Figure 2:**
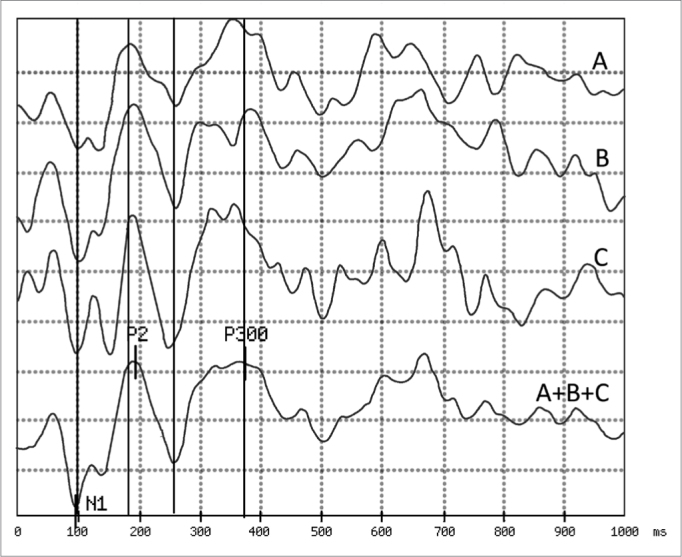
Example of a difficult exam to establish P300 latency: The first three traces (A, B and C) correspond to responses to 60 rare stimuli each, obtained in sequence from the same individual. Considering the instability of the responses obtained, the peak corresponding to the P300 can only be safely marked in the fourth trace (A+B+C) which corresponds to the algebraic summation of the first three.

To measure the P300 auditory latency we obtained a response by summation (made by the equipment software) of two or three sets of stimuli performed. Thus the measure of P300 latency used for the analysis in this study was the result of the sum of 120 or 180 responses to the rare stimulus, with great advantage in the correct definition of latency over the analysis of only 60 responses routinely employed in most studies. Figure 2 illustrates this approach.

In order to compare the P300 latency mean values in relation to age groups, we used an analysis of variance (ANOVA), with their respective tests for least significant difference (LSD), Duncan Test. In order to evaluate possible variations in P300 as a function of age we used the Linear Regression Coefficient. In all tests, conducted in SAS 8.02, the level of significance was 5%.

## RESULTS

Of the 253 elderly invited, 226 answered the hearing self-assessment questionnaire (Appendix A). Were excluded from the study 88 elderly with altered audiometries, beyond the accepted standards for this study, 36 who withdrew, 11 were not found, 07 who took antidepressants, 07 had P300 latency below 250 ms or greater than 500 ms, 06 who did not have P300, 03 who had had strokes, 01 who had chronic renal failure, 01 suffering from mild Alzheimer and 01 with mild sequelae from chronic otitis media. The groups were made up of elderly people aged 60 to 64 years (Group 60), 65-69 years of age (Group 65) and 70-74 years of age (Group 70), which were distributed as follows:

– Group 60: comprising 19 subjects 60-64 years of age, 16 females (84%) and 03 males (16%).
– Group 65: including 22 individuals aged 65-69 years of age, including 18 females (82%) and 04 males (18%).
– Group 70: made up of 21 elderly people 70-74 years of age, 16 females (72%) and 05 males (28%).

**Graph 1:**
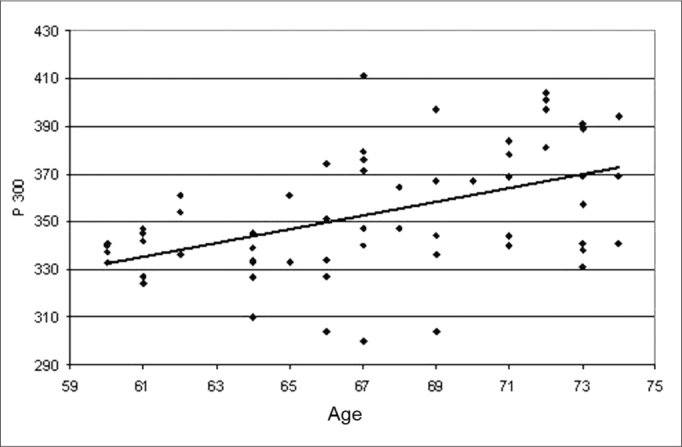
Scatter of the P300 latency values (in ms) in function of age (in years). The scatter analysis resulted in: P300 = 161,46497 + 2,8539* Age r2 = 0.2270 (p<0.0001)

The P300 latency values found in this study in the elderly, depicted on Table 1, showed a significant statistically proven trend to increase as age increased. These values were of 337 ms in the group 60-64 years of age with standard deviation (SD) of 11.31 ms, 351 ms with SD of 29.05 ms in the group 65-69 years of age and 370 ms with SD of 23.40 m in the group 70-74 years of age. These results are illustrated in Table 1. The linear regression model, shown in Graph. 1, depicted a significant increase under the point-of-view of statistics of 2.85 ms per year of age in the age group studied.

**Table 1:** Distribution of the groups as to the P300 latency variable

		gender		P300		
Groups	n	f	m	Mean	Standard deviation	Duncan test
60	19	16	03	337.26	11.31	c*
65	22	18	04	351.86	29.05	b*
70	21	16	05	370.19	23.40	a*

* p>0,0001

## DISCUSSION

In 1978, Goodin et al.10 were the first to show that the P300 latency varies according to age. They studied a group of 47 individuals with ages varying between 6 and 76 years, and only 07 had more than 60 years of age. In that study they used the same stimuli and the same task we used. They found a mean of 390 ms for the 60 to 75 years of age range. They stated that the P300 latency increased in 1.8 ms after 15 years of age. The latency calculated for 15 years was 294 ms (SD 21). Using this formula we would reach estimated values of 379 ms, 388 ms and 397 ms for groups 60, 65 and 70 respectively, which are higher values than the ones found in the present study. To explain this disagreement we state that the number of elderly they tested (07) was different from the numbers we had in our study (62).

In 1983, Brown et al.12 obtained 342 ms as P300 mean latency (SD 40.5) for a sample of 24 individuals between 45 and 80 years of age (mean of 65 years), a figure close to the one we found (337 ms in the 60 to 64 years of age group). For 25 individuals below 45 years of age they found the value of 304 ms (SD 22). The direct comparison of the findings from this study can not be carried out because the age range they studied was much broader (45 to 80 years) and the number of individuals was lower (25) when compared to what we had in our study (age range between 60 and 74 years and 62 individuals).

In 1983, Polich and Starr13 obtained a mean latency value for the P300 of 354 ms (SD 35) in a sample of 36 individuals with more than 45 years of age (mean of 59.7) near the value of 351ms found in this study in the group of 65 to 69 years of age. The group of adult individuals with less than 45 years of age had a mean P300 value of 310 ms (SD 24.7), clearly inferior to that of the older group.

In 1982, Syndulko et al.18 found the mean latency value for the P300 of 368 ms (SD 32.1) in a sample of 25 individuals with more than 45 years of age (mean of 63) similar to the value of 370 ms found in this study in the group of 70 to 74 years of age. The value of 330 ms (SD 27. 2) was seen in 20 individuals with less than 45 years of age (mean of 29).

Nunes19, in the first Brazilian study involving the elderly, studied a sample of 30 normal senior citizens between 65 and 75 years of age, found the P300 latency in 363.07 (SD 37.08). Gathering groups 65 and 70 in this study (N 43) we found a mean of 360.81ms (DP 27.7) showing agreement between the two studies.

Matas et al.20 examined 24 individuals aged between 50 and 79 years. The individuals were broken down in three groups: G1 (50 to 59 years), G2 (60 to 69 years) and G3 (70 to 79 years). P300 showed a mean latency of 331.71 ms in G1; 370.67 in G2 and 407.50 for G3, which were statistically significant. Joining groups 60 and 65 (N 41) we obtained in this study a mean value of 344 ms. Such value is statistically different from the one obtained by Matas et al.^20^(370 ms). The number of individuals studied was much lower (08 per group), the frequency of the rare stimulus used was 1500 Hz and the stimulation was monaural instead of binaural, which must be the cause for this discrepant results.

In a general analysis, this study agrees with the literature data showing higher latency values obtained for the P300 in older adults than those obtained from young adults. In studies that showed different results^11^, ^12^, ^13^, ^14^, ^15^, ^16^,^20^ the very small number of individuals examined should be the main explanation for the differences found. There is agreement among all studies and the present one on the observation that the older the individual, the higher the P300 latency. Polich14 noticed in a meta-analysis of different studies on the speed with which the change of P300 latency occurs in different age groups, from childhood to old age, the frequency of rare or frequent stimuli to be a relevant factor in the outcome of measures, considering that each frequency used as input evaluates various topographical areas of the auditory pathway. By the same token, the task clearly interferes with the latency values obtained, which are larger when employing the counting of rare stimuli than when they have to press a button.

The use of other types of speech sound stimuli, soundless intervals, a single-frequency tone with a variable inter-stimulus interval and others add more variables to establish reference values for the P300 latency values on the different age ranges.

We noticed that the type of stimulus and task employed in this study is in agreement with most of the authors. The use of other types of stimulus and/or task found in the literature can be used to show that changes in these protocols lead to different results. It is suggested that we must maintain the same type of protocol employed in this paper to other P300 studies.

It is believed that with the recent developments in the field of CAP disorders diagnosis and treatment, the study of the P300 in the elderly population with hearing complaints associated with speech understanding in environments with sound competition which are not explainable through its audiograms can bring about unique and important information.

By better understanding the electrophysiological substrate associated with the central auditory dysfunction and the possibility of following on the treatment results with a quick test (around 30 minutes) and of low cost when compared to the behavioral tests used to assess central auditory processing are aspects which must be stressed.

The results of our study confirm that latency values increase with age – which reinforces the idea that this variable must be taken into account when interpreting P300 results.

The results from the present study can be used as reference values for other studies which can be done in groups suspected of having CAP disorders.

In order to characterize the maximum latency considered normal for the ages studied, we recommend the mean value obtained added to two standard deviations, namely:

– age range between 60 and 64 years: 360 ms
– age range between 65 and 69 years: 410 ms
– age range between 70 and 74 years: 420 ms

## CONCLUSION

– An age increase is followed of a statistically significant increase in the P300 latency on the age range studied.
– This increase happens linearly with a statistically significant increase of 2.85 ms per year in the age range assessed.
